# Simple method for distinguishing the intersegmental plane in thoracoscopic lung segmentectomy

**DOI:** 10.1111/1759-7714.13417

**Published:** 2020-04-06

**Authors:** Peng Jiao, Yaoguang Sun, Wenxin Tian, Qingjun Wu, Hongfeng Tong

**Affiliations:** ^1^ Department of Thoracic Surgery, Beijing Hospital, National Center of Gerontology Institute of Geriatric Medicine, Chinese Academy of Medical Sciences Beijing China

**Keywords:** Intersegmental plane, segmentectomy, video‐assisted thoracoscopic surgery

## Abstract

Here, we introduce a simple method for delineating the intersegmental border in thoracoscopic pulmonary segmentectomy which can be widely reproduced because it is less time‐consuming, fault‐tolerant, and does not require any special chemical reagents or equipment. This method provides clear and accurate demarcation lines between the inflated and deflated lung parenchyma.

**Key points:**

A method with an effective and simple application which can be popularized. This modified targeted bronchus inflation method provides a clear and accurate intersegmental plane.

## Introduction

Many methods^1^, ^2^, ^4^, ^5^ with their pros and cons have been previously reported which demarcate the borders of the segments, but none have been accepted by most surgeons until now. The aim of this study was therefore to report a new simple and practicable method of intersegmental plane demarcation.

## Methods

### Data

**Figure 1:**
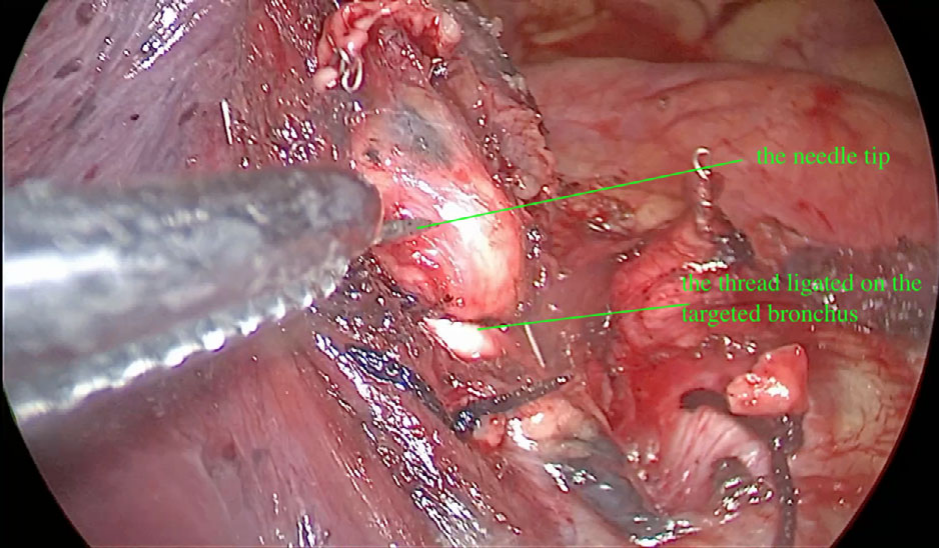
The moment before a needle was punctured into the targeted bronchus.

**Figure 2:**
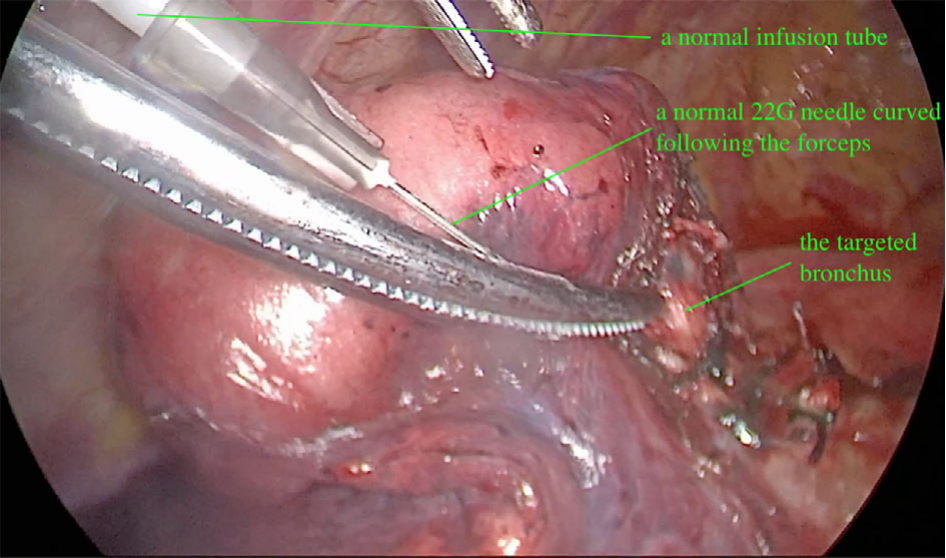
This shows the moment that air was insufflated into the bronchus and the lung parenchyma inflated.

We retrospectively analyzed the data of 167 patients who underwent pulmonary anatomic segmentectomy between May 2017 and January 2020. The method was used for 96 segmentectomies in 92 patients, as two segments were resected in the same procedure in four patients. A total of 35 males and 57 females were included in the study, with a mean age of 63.9 years (range: 38–85 years). The inclusion criteria were as follows: (i) patients with clinical stage I non‐small cell lung cancer (NSCLC) who could tolerate operative intervention but not lobar resection because of decreased pulmonary function or comorbid disease; and (ii) a tumor diameter less than 2 cm indicated on computed tomography (CT) scan and morphologically diagnosed as pure ground‐glass (GGO) or mixed GGO (GGO: >50%) (Figs 1, 2).

### Surgical procedure

All the operations were performed using two‐port video‐assisted thoracoscopic surgery (VATS). After endotracheal intubation, in a state of unilateral differential ventilation, after the targeted segment artery and vein were dissected by ligation or stapler cutting, the involved bronchus was identified and ligated with a normal 7# silk thread on the proximal part. A needle (range: 20–23 G) connected with a normal infusion tube, was curved just according to the forceps, and punctured into the distal part of the bronchus. The needle tip was kept beyond the forceps about only 4–6 mm, as it should be shorter than the diameter of the targeted bronchus to avoid penetrating it and causing accidental injury. A further procedure to make sure the needle tip was in the bronchus instead of blood vessel was to draw air as opposed to blood out though the infusion tube. Oxygen or air flow (1–3 L/minute) was then used to insufflate the targeted lung segment parenchyma, and once inflated, a clear line between the inflated and deflated lung parenchyma appeared. The targeted segment was divided using a stapling device or cautery, depending on the surgeon's preference, and removed.

## Results

**Figure 3:**
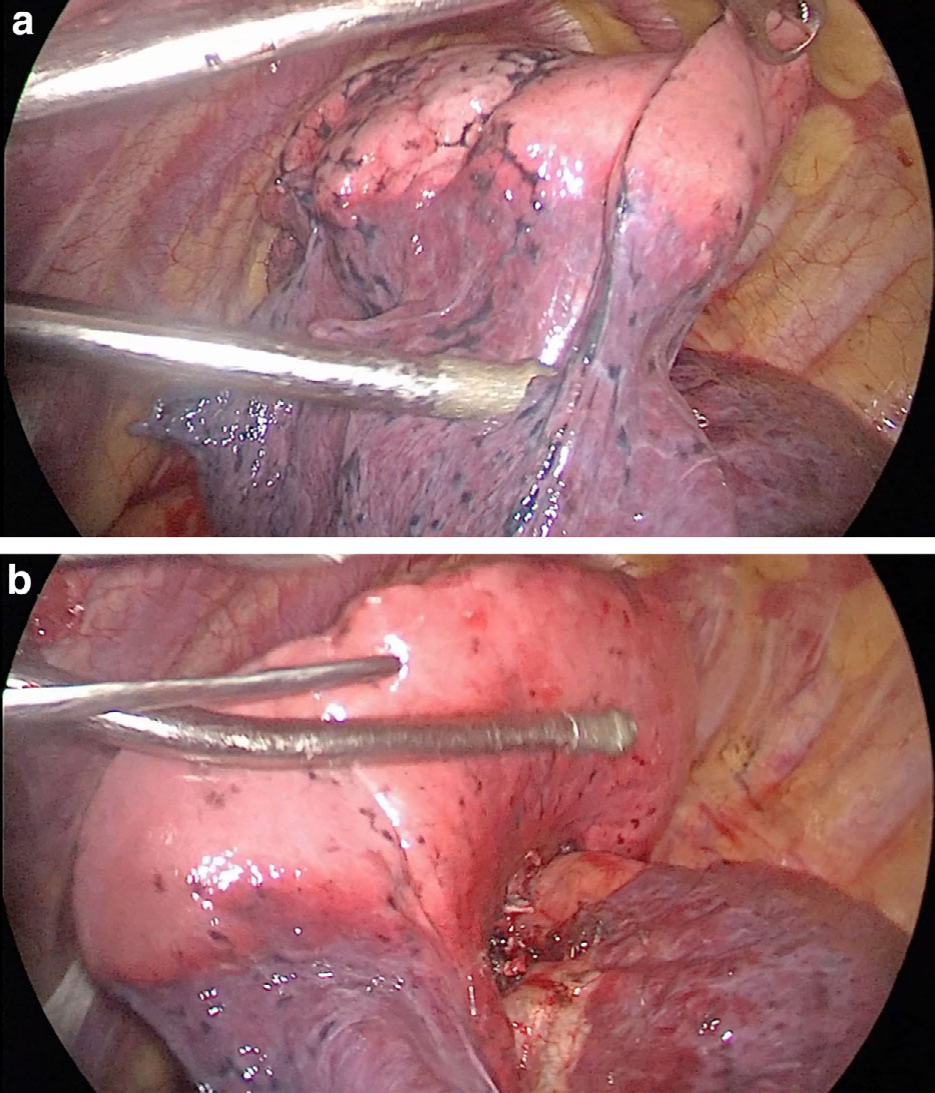
(**a**,**b**) There is a clear demarcation between the deflated and inflated lung segment.

All the operations using this method went well, and no deaths or serious complications occurred in the perioperative period. Following insufflation of oxygen or air, a distinct demarcation between the targeted and other segments appeared instantly in 85 cases. However, in 11 cases, not only the targeted but also the adjacent segmental parenchyma inflated at the same time. To save operation time, other operative procedures such as lymphadenectomy could be undertaken because there would be more space for instrument manipulation with only one inflated lobe. About 10 minutes later, a satisfactory demarcation was observed in all 11 cases. At the time of insufflation of oxygen, the wrong segment inflated in two cases. Fortunately, the bronchus had not been severed, and therefore the thread ligated on the bronchus was unfastened. The correct bronchus was operated more carefully. No pulmonary atelectasis or other complications occurred in these two cases (Fig 3a,b).

## Discussion

There have been several similar methods previously reported with a range of pros and cons. For example, the slip‐knot method^5^ first requires bilateral ventilation, and the knot is then tightened to block the outflow of segmental air. All pulmonary parenchyma will inflate, which occupies too much space, so that the operation has to be halted to wait for the lung to deflate. The jet ventilation approach^6^ requires the use of a flexible bronchoscope where it is impossible to match exactly all the targeted segmental bronchi, and air may leak into the adjacent bronchi, leading to an unclear or inaccurate line. Further, oxygen cannot be insufflated when the bronchoscope is approaching the targeted bronchus, and often it is not easy to approach the segmental bronchi due to changes in the angles of subsegmental bronchi during respiration and for anatomical reasons. The butterfly needle method^7^ is the most similar to ours, but in that study the bronchus was first stapled, and the segment inflated with a butterfly needle. We ligatured, but did not dissect the bronchus which commonly varies as known to all, in case the wrong bronchus was divided.

This modified targeted bronchus inflation method which does not requires any chemical reagents (such as indocyanine green) or equipment (such as an endoscopic fluorescence imaging system or bronchoscope)^2^ is very simple with no additional expense which is important as there are numerous patients in China. Dissection along the plane between the inflated and deflated lung parenchyma using either electrocautery or staples is safe, with almost no air leakage or bleeding, which means the demarcation provided by the method is highly coincident with the real intersegmental border. This method is also less time consuming because the time it usually takes for inflation‐deflation to take place is saved. Even if the perfect demarcation does not appear at once due to inflation of the adjacent segmental parenchyma, other surgical procedures can be performed because only one lobe has inflated and would not occupy as much space as traditional or other modified inflation‐deflation methods.^3^ The method is fault‐tolerant and safe. Instead of dissection, the bronchus is ligated first to prevent division of the wrong bronchus.

In conclusion, this new method provides a clear and accurate intersegmental plane, which simplifies lung segmentectomy without any additional reagents or equipment. It can reduce the duration of operation and anesthesia, and facilitate a safer and fault‐tolerant procedure. Additionally, it is no more expensive than any other methods, is easy to learn, with an effective and simple application which can be popularized.

## Disclosure

The authors declare that they have no conflicts of interest.
